# Automated Microclimate Regulation in Agricultural Facilities Using the Air Curtain System

**DOI:** 10.3390/s21248182

**Published:** 2021-12-08

**Authors:** Nikolay Kiktev, Taras Lendiel, Viktor Vasilenkov, Oksana Kapralуuk, Taras Hutsol, Szymon Glowacki, Maciej Kuboń, Zbigniew Kowalczyk

**Affiliations:** 1 Department of Automation and Robotic Systems, National University of Life and Environmental Sciences of Ukraine, 03041 Kyiv, Ukraine; taraslendel@gmail.com; 2 Department of Intelligent Technologies, Taras Shevchenko National University of Kyiv, Volodymyrs’ka Str., 64/13, 01601 Kyiv, Ukraine; 3 Department of Heat Power Engineering, National University of Life and Environmental Sciences of Ukraine, 03041 Kyiv, Ukraine; wasil14@ukr.net; 4 National Scientific Agricultural Library of the National Academy of Sciences of Ukraine, Str. Mikhail Omelyanovich-Pavlenko, 9, 01010 Kyiv, Ukraine; Oksana_Vasilenkova@ukr.net; 5 Department of Mechanics and Agroecosystems Engineering, Polissia National University, Stary Boulevard, 7, 10008 Zhytomyr, Ukraine; pro-gp@pdatu.edu.ua; 6 Department of Machine Use in Agriculture, Dmytro Motornyi Tavria State Agrotechnological University, B. Khmelnytsky Ave., 18, 72312 Melitopol, Ukraine; 7 Institute of Mechanical Engineering, Warsaw University of Life Sciences-SGGW, 02-787 Warsaw, Poland; glowackisz@gmail.com; 8 Department of Production Engineering, Logistics and Applied Computer Science, Faculty of Production and Power Engineering, University of Agriculture in Kraków, Balicka 116B, 30-149 Kraków, Poland; maciej.kubon@urk.edu.pl (M.K.); Zbigniew.kowalczyk@urk.edu.pl (Z.K.); 9 Eastern European State College of Higher Education in Przemyśl, Książąt Lubomirskich 6, 37-700 Przemyśl, Poland

**Keywords:** thermal air curtain, microclimate, gates, energy saving, automation, control, integrated board, SCADA-system

## Abstract

Creating and maintaining the microclimate in livestock buildings is associated with numerous engineering and technical challenges. Together with adequate feeding, the microclimate determines the health, reproductive ability, and production potential of the animals (obtaining a maximum amount of high-quality products). One of the deciding steps in improving the parameters of microclimate, i.e., temperature and humidity in agricultural facilities, particularly in livestock buildings, is to develop reliable and highly efficient air curtains in the vestibules. The objective of the manuscript is to investigate the parameters of the microclimate in livestock buildings using the air curtain, supported by automation and ICT technologies for rational operating modes. The presented theoretical and experimental studies on improving the microclimate parameters in livestock buildings were carried out using an innovative air curtain system. Its power is calculated based on the dimensions of the room, and the flow rate of warm air near the floor level is three times lower than at the installation site. The use of air curtains reduces consumption of thermal energy needed to maintain an optimal microclimate for livestock by 10–15%. Furthermore, the use of an automated digital control system maintains an optimal microclimate in the building. The developed energy-saving system for creating an optimal micro-climate in livestock buildings using air curtains was tested in a pigsty of the Research and Training Farm “Vorzel” of the National University of Life and Environmental Sciences of Ukraine, located in the Kiev region. The developed automated microclimate system using air curtains significantly improves the microclimate parameters and significantly reduces power consumption. The system can be further developed by adding remote control based on the Internet of Things (IoT) technology.

## 1. Introduction

Modern animal husbandry technologies place high demands on the microclimate in livestock buildings. According to animal husbandry experts, the productivity of animals is determined by feed in 50–60%, by care in 15–20%, and by the microclimate in the livestock room in 10–30%. Deviation from established limits of microclimate parameters leads to a reduction in milk production by 10–20%, the consumption of additional feed followed by an increase in live weight by 20–33%, an increase in the waste of young animals by 5–40%, a reduction in the laying of chickens by 30–35%, and reduced immunity, as a well as shortening of the life of equipment, the machine park, and buildings themselves [1,2,3,4,5].

Ensuring acceptable conditions for efficient operation of a livestock farm in winter, especially maintaining normal temperature, requires considerable resources. However, the efforts to insulate the walls and install a proper heating system can be futile if heat leaks freely through windows or gates. This is especially true of livestock buildings in which the front gate is opened very often, or even remains open for an extended time. This challenge is addressed by installing an air curtain on the front door in livestock vestibules. Therefore, one of the important directions of the agenda for the conservation and efficient use of fuel and energy resources is the development and implementation of energy-saving equipment to create a microclimate in livestock buildings. This means not only increasing the technological capabilities of air curtains, but also introducing automated control systems to ensure that process parameters are maintained at the set level.

The purpose of this study is to improve the functioning of the microclimate in livestock buildings through the use of an air curtain, to determine its rational operation, and to introduce an automated control system using the proprietary mechanism based on an integrated Arduino board, along with a control system using the LabView programming language. We hypothesized that the use of air curtains supported with automation reduces the consumption of thermal energy to maintain an optimal microclimate in livestock buildings by at least 10%.

Research in this area is carried out in different countries (Slovakia, Russia, Ukraine, etc.) as it can offer a comprehensive remote microclimate monitoring and control system for a livestock building using IoT and low-code software deployment. The stakeholders of this project are both livestock holdings and farms, since there is a shortage of quality food products in the world, and Ukraine (along with Russia and Belarus) in recent years have become major food exporters.

## 2. Literature Review and Problem Statement

Livestock is one of the main power consumers in agriculture, with a share of 17.2–21.3% in the total power consumption of the industry. Furthermore, cattle farms are the main energy consumers in animal husbandry (accounting for 46–51.5% of the total industry power consumption) [1].

Following an analysis of the structure of power consumption for milk production, it was determined that the highest share in the total costs is the power consumed to create and maintain an optimal microclimate in livestock buildings (Table 1). Its share, depending on the husbandry technology, is 34.5–36.8% [1,2,3,4,5], which can be compared only to the power consumption in the preparation of feed mixtures. Therefore, one of the main directions of reducing the total power consumption for milk production, and therefore its total cost, is the development and implementation of energy-saving equipment to create and maintain a recommended microclimate in livestock farms.

One of the most promising and relevant areas of energy conservation in microclimate support systems is the use of air curtains, which reduces the consumption of thermal energy to maintain an optimum microclimate in livestock buildings by 10–15% [1]. However, these works do not offer statistical processing of experimental data; the thermal energy is calculated based on a deterministic model based on the average temperature difference in the cold season (−5 °C outside and 20 °C inside the room). The authors believe that it is necessary to consider other temperature options to justify the choice of the air curtain’s power and achieve an efficiency of 10–15%.

The problems of numerical simulation of hydrodynamics and mass transfer processes for an effective location of the ventilating system were solved by researchers from the National University of Life and Environmental Sciences of Ukraine [6,7,8]. The authors calculated the optimal location of fans and water-cooling systems in poultry houses. These articles are a significant contribution to the development of microclimate technologies for agricultural facilities, but they do not address the issue of automating the maintenance of microclimate parameters.

The problem of optimizing energy conservation in a livestock building was posed and solved in [6,9]. Improved methods for solving multicriteria optimization problems are described in [10].

Nalivaiko, V., Radko, I., Zhyltsov, A. et al. [11] investigated the microclimate of buildings subject to thermal renovation and using renewable energy. In this research, the rooms with the lowest and highest temperatures were identified. The subject of study was one of the university buildings, in which the cause of uneven temperature distribution was investigated. The article presents the possibility of leveling the distribution of internal air temperatures and reducing the dependence of their values on fluctuations in the outside air temperature. The authors demonstrated the energy efficiency of intermittent heating, which allows reducing thermal energy consumption. To decrease the energy costs, the authors suggested using a heat pump that compensates for peak loads and operates at a discount rate at night. These studies can also be applied to a livestock building to improve its energy efficiency. However, agricultural buildings have their own specifics, which is not taken into account in this manuscript. In addition, these studies do not include software and hardware automation tools, which enable effective management of renewable energy sources.

In an article by researchers from the Federal Scientific Agroengineering Center “VIM” (Moscow, Russia), Tikhomirov, D., Vasilyev, A.N. et al. investigated the automation of the microclimate of agricultural facilities using air ventilation [12]. The structural and functional diagram of a ventilation and heating installation with heat recovery, ozonation and air recirculation was presented. The automation system is based on OWEN devices, especially OWEN UKT-38, the eight-channel meter with alarm, and a thermostat OWEN TRM-138. OWEN AS-2/AS-4 interface converters are used to communicate with the computer. Two humidity sensors and eight thermocouples were selected as measuring instruments. The system software is represented by the OWEN standard product, Owen Process Manager (OPM), as well as device configurators, which are sufficient for a simple control system. The correlation dependence of the heat flux returned by the heat exchanger and the difference between the external and internal temperatures of the room was experimental and calculated using the model. The article also offers a graph of temperature changes during the day at different phases of the automated system. The OWEN line of devices is admittedly a powerful automation tool that allows, e.g., to remotely monitor the progress of the process in the OWENCloud environment. However, the use of the software is limited to the standard OPM and OWENCloud interface, which limits the development of the system, remote control capabilities, and upgrading the system interface. In our paper, the authors offer a more flexible interface based on the LabView package, which can be easily upgraded to add other devices, functionalities, as well as calculation and processing capabilities.

Švajlenka, J., Kozlovská, M., Pošiváková, T. from Slovakia described the monitoring of agricultural facilities for protection from biological factors, especially pests that spoil agricultural products and destroy the structure of the building [13]. Microbes contained in the building, e.g., mold and other microorganisms, negatively affect the health of animals, the quality of stored vegetables, and also affect the formation of rust and the subsequent destruction of the building structure in the medium term. The researchers suggest indoor air sampling and biological analysis. These authors presented a deeper analysis of the influence of the internal and external environment of agricultural buildings [14]. In particular, the article offers a study of the impact of the environment and internal microclimate on the health and productivity of animals. The graph of heat transfer on the wall in winter during the day was presented, as well as the presence of microorganisms of two types (*Cladosporium sphaerospermum* and *Fusarium graminearum*) in the indoor air during the studied seasons, summer and winter.

The above-mentioned works can be used in the future for the development of the project described in this manuscript. However, they do not address the issues of automated control of the microclimate of agricultural buildings. On the other hand, literature offers research on using integrated Arduino boards and developing a LabView-based SCADA system to control ash content in a coal mine and to monitor the environment [15,16]. The control system for agricultural production discussed in this manuscript is also based on an integrated Arduino board, but additionally uses IoT technology [17]. These projects discuss the automation of similar areas, but they are suitable for consideration of technology described in this article.

The research on the subject of this article was carried out by Shelekhov, I. Yu., Dorofeeva, N.L., Smirnov, E.I. et al. [18], engineers from Irkutsk (Russia). The authors researched new innovative methods of creating the most favorable indoor temperature, and the impact of a new type of infrared air curtains on the penetrating cold air currents. Experiments have shown that the best microclimate is achieved with a double-flow system consisting of a heated air curtain and a conventional air curtain.

On the other hand, Tikhomirov, D.A., Trunov, S.S., Kuzmichev, A.V. et al., scientists of the Federal Scientific Agroengineering Center “VIM” (Moscow, Russia), developed an energy-efficient thermoelectric unit for microclimate control in cattle breeding barns [19,20]. The authors studied the features and the possibility of using the original model to calculate the required power of air curtains for agricultural facilities, and demonstrated the most promising solutions for air curtains and the main directions for their improvement. They confirmed that the share of the impact of microclimate on the productivity of animals is 25–30%. Their modular air curtain protects door openings with a variable air flow vector and an adjustable width, mixed-type. It is installed horizontally in the gate of a building, with an upper air supply, or vertically with a one-way or two-way air supply. The device includes a heater, a fan, air ducts, an air intake switching valve, and an air diffuser located vertically or horizontally in relation to the opening (depending on the opening type).

The above studies are the most relevant to this research, as they relate to air curtains for livestock buildings in both theoretical and practical aspects. Although they discuss technological aspects and offer deterministic models, they lack the presentation of the processed results of experiments, followed by a proposed answer to optimization problems, as well as hardware–software solutions to automate the process.

On the other hand, Korean scientists Kim, H.K.; Kang, G.C. et al. investigated the thermal insulation of a greenhouse using the air curtain [21] to ensure the required temperature. It has been proven that installing air curtains is an effective way to conserve heat, and using them in greenhouses at night will increase thermal insulation and conserve energy by 28.7%.

In a research by Egyptian scientists Akrami, M., Mutlum, C.D. et al. [22], a computational fluid dynamics (CFD) model of a solar powered desalination greenhouse was developed. To analyze the microclimate in the greenhouse, wrap patterns, air streams and temperature boundaries were identified. The greenhouse design parameters have had a significant impact on the air distribution. In the investigation, two scenarios were considered: “the bottom vent hole is open” and “both vent holes are open”, while the formation of vortices around the upper left corner is visible on the temperature contours. The presented model can be used to create a sustainable, self-sustained greenhouse with natural ventilation, suitable for countries facing water and power shortages. These studies can affect not only the microclimate (wind speed and temperature) inside the greenhouse but also its design.

Strongin, A.S. and Zhivov, A.M. (USA, Russia) [23] describe energy-efficient air curtains for industrial and warehouse facilities in a cold climate. The article provides an assessment methodology that can be used to determine the application of different types of air curtains. In addition, the work of Safarzadeh, M., Heidarinejad, G., Pasdarshahri, H. (Iran) [24] is of interest; the authors propose using an air curtain in a multi-story building to control smoke and heat. This compares vertical and horizontal air curtains. It was found that the vertical air curtain does not work well in an insufficiently ventilated enclosure, while the horizontal air curtain acted as a fire regulator and retained the mass fraction of toxic substances (CO and CO_2_).

A team of British and Italian scientists led by Foster, A.M., Swain, M.J. and Barrett, R. [25] investigated the three-dimensional effects of the air curtain that limit the penetration of air into a cold room, which is important for refrigerated warehouses. Reducing the deflection of the edges of the air curtain will increase its efficiency. 

The above works also relate only to technological aspects, excluding hardware and software for automation of the process, which would allow monitoring and managing the microclimate of the room more efficiently.

Researchers from Ukraine and Poland [26,27] have developed a forecast software based on modeling risk assessment in biohydrogen as a source of thermal energy from agricultural raw materials. An important component for ensuring the temperature regime in agricultural buildings, including livestock buildings, is the building material. To prevent moisture permeability of the wooden elements of the room, they are subject to thermal renovation, as investigated in a number of publications by Mazurchuk, S., Marchenko, N., Tsapko, Y. et al. [28,29,30], researchers from Ukraine and Slovenia. On the other hand, the research on the thermodynamic properties of a room and the construction of thermodynamic models was carried out by researchers from Ukraine and Slovakia, Pinchevska, O., Spirochkin, A. et al. [31,32,33,34,35]. The proposed models were tested for the homogeneity of the mean values and dispersion of the current moisture content, which showed a slight difference between the experimental values and the calculations. The constructed models allow predicting the quality of the technological process based on the distribution of the moisture content, which allows choosing rational graphs of the temperature and humidity conditions in the room. 

The literature offers mass transfer control models for various thermodynamic systems [36]. The work of Ukrainian researchers explores the efficient autonomous power supply of agricultural holdings, in particular the wind power model. It is based on stochastic modeling and provides cost justification for the parameters of this system [37]. The application of cluster analysis and forecasting methods for the effective use of renewable energy sources in the power supply of an agricultural holding was studied in [38]. This work discusses the development of applied mathematical and software-based support (forecasting, cluster analysis, and stochastic modeling) for heat exchange processes and controlling renewable energy sources in various technological facilities. These studies are also useful for the point of view of this research, both from a technological and from a mathematical point of view. In particular, distributed cloud-based systems can be used for remote control of the microclimate using IoT, whereas forecasting and clustering methods can be used to create an integrated information and control system for the microclimate. 

## 3. Research Objective

The purpose of the research is to improve the functioning of the microclimate in livestock buildings by using the air curtain, to determine the rational modes of its operation, and to introduce an automated control system using information and communication technologies. 

Research goals are as follows:

- analyze domestic and foreign trends in improving technologies, hardware, and software for microclimate control in livestock buildings;
- to generalize and establish regularities of the impact of microclimate parameters on power consumption; and
- to conduct experimental studies and field tests of the air curtain in existing farms.

The research objective is to optimize the parameters of technological processes and their mechanization, as well as hardware and software automation. The research object is a set of microclimate parameters (humidity, as well as external and indoor temperatures at different points in the room), as well as technology and technical means to ensure the microclimate. 

## 4. Materials and Methods

Theoretical and experimental studies on improving the microclimate parameters in livestock buildings were carried out based on a schematic electrical diagram of an air curtain, as well as statistical methods for processing experimental data, using LabView-based software and automation hardware based on the Arduino controller.

**Figure 1:**
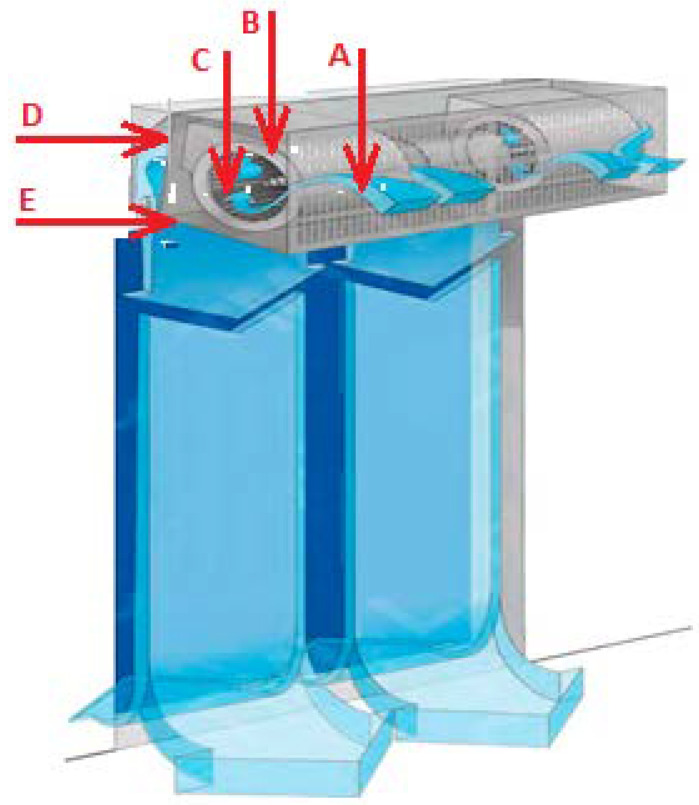
Principle of operation of the air curtain. A—front grille; B—fan baskets; C—injection wheel; D—air chambers; E—outlet duct.

The main function of the air curtain is to maintain the temperature in the room at the required level that is comfortable for both the livestock and the farm staff. For this reason, an air curtain is usually considered a heater, although the principle of its operation is not to heat the air in the room but to cut off the flow of air from the outside. An air curtain creates an invisible barrier for separation and environmental management. The surrounding air space passing through the front grille (A) is sucked into the fan baskets (B). The injection wheel (C) directs the flow to the air chambers, compressing it (D), and blows it down through the outlet duct (E), creating “invisible door” that serves as a barrier (Figure 1).

Air curtains are installed in different parts of buildings, taking into consideration the following factors:

1. At gates that open more than 5 times or for at least 40 min per shift, located in areas with an estimated indoor temperature of −15 °C and below in the cold season.
2. At gates or openings, at any outside temperature and at any opening time, as deemed justifiable.
3. In the lobby and aisles near the entrance doors to the lobby of the public and auxiliary buildings of industrial plants.
4. In lobbies and aisles at the entrance doors of public and industrial buildings and premises with air conditioning systems.

Air curtains should ensure that when the door or gate is opened in a workplace, indoor temperature is 14 °C or higher during light work, 12 °C or higher during medium work, and 8 °C or higher during heavy work. In the absence of a door, the minimum temperature can be reduced to 5 °C in a workplace, and in lobbies of public buildings to 12 °C. The curtain air temperature is generally below 50 °C. The velocity of the air outlet of the air curtain is no more than 25 m/s (in industrial buildings). 

The air curtain model is selected based on the required power. The main parameters for the calculation included the width and height of the inlet/outlet slot; the length of the curtain air stream; voltage (220 or 380 V); efficiency and thermal capacity of the device; as well as indoor and external air temperature. 

Step-by-step selection of an air curtain for a vestibule of a livestock room with a heated door opening 3 m wide, 3.5 m high is as follows:

1. The air curtain power is calculated using the formula:

(1) N=V⋅ΔT⋅K, kcal/h

where:

*V*—the volume of the vestibule for which the air curtain is selected (width 9 m, length 4 m; width, height of the door opening 3 and −3.5 m, respectively); the calculated volume of the vestibule = 126 m^3^;

∆*T*—the temperature difference (taking into account the external temperature, e.g., −20 °C and the desired internal temperature in the vestibule, e.g., +5 °C; the calculated ∆*T* = 25);

*K*—heat loss coefficient (calculated according to Table 2);

*N*—required power, determined in kcal/h; the resulting number should be subsequently converted to kW at 1 kW = 860 kcal/h.

**Figure 2:**
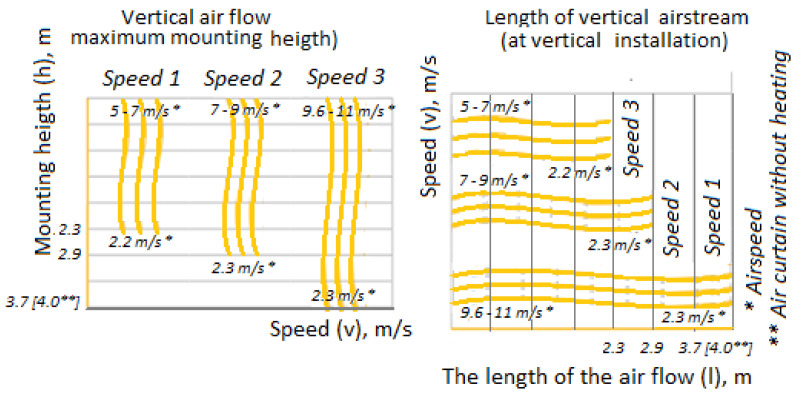
Compliance of the length of the air stream with the height (width) of the door.

2. The length of the air curtain shall be equal to, or greater than the width of the door opening by 10%, selected according to Figure 2.
3. The length of the air stream must correspond to the height (width) of the door. For example, at a curtain height of 2.3 m, the velocity of the air stream is approximately 5–7 m/s at the outlet of the air curtain and approximately 2.2 m/s at the floor level; in the studied case, at an installation height of 3.7 m at the outlet, velocity of the air stream is 9.6–11 m/s, and at the floor level, 2.3 m/s. If the length of the air stream is insufficient, the air curtain will be inefficient, immediately increasing the heat losses.
4. The main requirement for the effective operation of the air curtain is the air velocity at the floor, which should be approximately 2 m/s (Figure 2).
5. The efficiency, heat output, and model of the air curtain shall be selected according to the specifications provided by the manufacturer.

To calculate energy consumption when using the air curtain, experimental measurements of the external and indoor temperatures in different parts of the vestibule of the room were carried out and calculations were carried out according to the formula (1). The results were subject to statistical analysis and are presented in Table 2.
Expected value1.450919865RMS2.174349597

**Figure 3:**
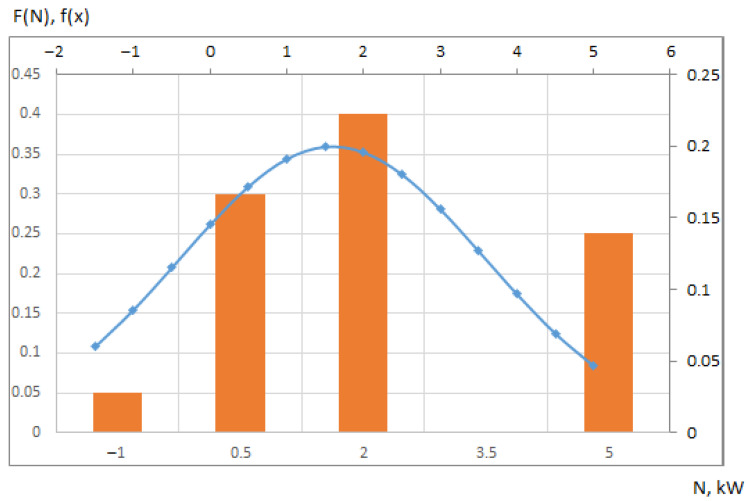
Energy consumption histogram and normal distribution function. N—calculated power; F(N)—frequency of a certain power drop in a given interval (orange columns); f(x)—the probability density curve of normal distribution (blue line).

The histogram and normal distribution function, prepared using the MS Excel package, is shown in Figure 3. The columns show the frequency of a certain power drop in a given interval F (N). The probability density curve of the normal distribution is shown by a blue line.

It was previously demonstrated [5] that to increase the energy efficiency of a livestock building by 10–15%, it is necessary to use an air curtain with a capacity of 4 kW. Thus, the authors tested the null hypothesis that the average value of the obtainable power is *µ* = 4. For verification, the authors used a *z*-test, in which the number of sample data was *n* = 30 [39].

(2) $$z=\frac{\left(\bar{x}-\mu \right)}{s/\sqrt{n}}$$

The authors used the *z*-test function of the MS Excel package. The result shows the probability that the power of the selected 4 kW air curtain will satisfy the design power N1 (the last column of Table 2) at any temperature ranging from −20 to 33 °C (most typical in Ukraine). The result of the test: *z* = 1 proves the hypothesis that the selected power fully satisfies the discussed purpose. At lower values of the air curtain power, the probability is less than 1, and at a power of 1 kW, *z* = 0.05, that is, the use of an air curtain of this power is impractical.

To measure the parameters of the microclimate, a temperature and humidity sensor DHT22 was selected. The ZD50 throttle valve was chosen as the regulating body, to operate the digital system of the controller of the hardware platform Arduino.

LabView software uses a number of libraries to communicate with the computer and external devices. For this purpose, the Virtual Instrument Software Architecture (VISA) tool is used, which is a standardized input/output interface widely used for testing, measuring, and controlling devices with a desktop computer. VISA supports interfaces such as IEEE-488 (GPIB), VXI, RS-232, as well as USB for measuring devices and is implemented (as e.g., NI VISA) as a library of functions for the C, Visual Basic, and G languages. This allows VISA to standardize access to all measuring devices, regardless of the protocol and hardware used (for example, regardless of the GPIB adapter model). Before implementing the Arduino controller management program project in LabView, the controller was pre-programmed with VISA interface commands. The Arduino Uno controller is programmed in the Arduino IDE environment using the C programming language. Then, the controller connected to the PC will accept the command protocol in the VISA interface in the LabView environment. 

**Figure 4:**
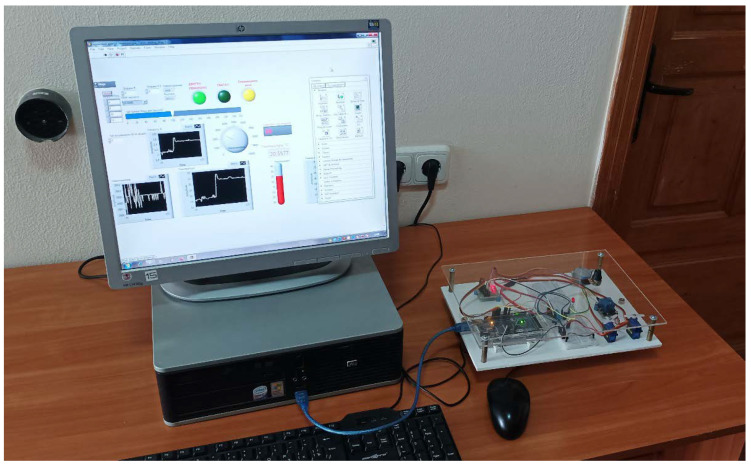
Pilot installation for the air curtain control based on an integrated Arduino board.

**Figure 5:**
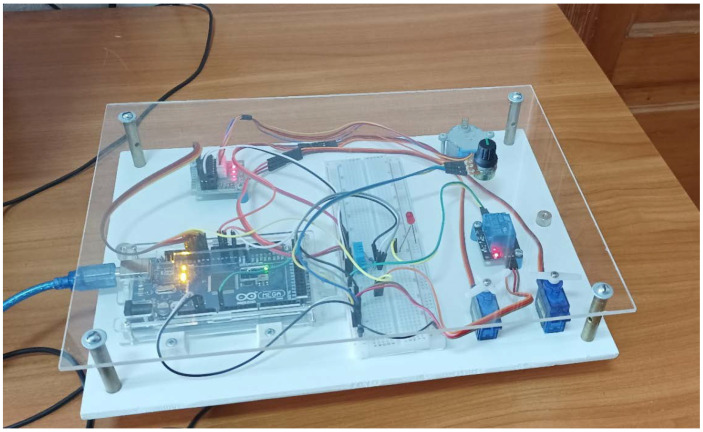
The hardware set.

A pilot installation for controlling an air curtain based on an integrated Arduino board is shown in Figure 4. A set of hardware based on the Arduino board and the sensor DHT22 and the throttle valve ZD50 is shown in Figure 5.

The system operation algorithm compares the value measured from the sensor (input–output unit) with the set boundary values of temperature (in this case 8–12 °C). On the other hand, the control launches the actuator of the air curtain. The READ block is intended for reading data from the sensor, the WRITE block is for saving the command buffer. Arduino contacts take on the following values: 2, when connecting an actuator; 4, when connecting a sensor. 

**Figure 6:**
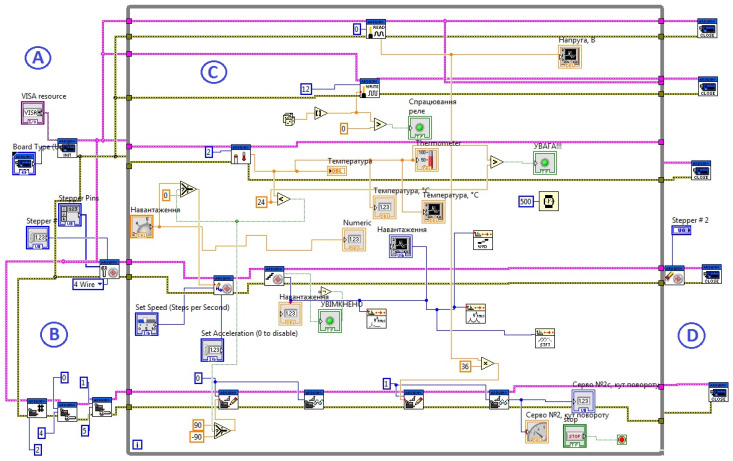
Control system interface. A—area for connecting external devices; B—input area for initial parameters; C—execution zone of the program logic in a cycle, input/output of digital and graphic information to the panel; D—execution zone of the measurement processes.

Using NI LabView software, a SCADA system was developed, the block diagram of which is shown in Figure 6. This software includes an interface, which provides automated control of the technological process and displays measured values and signals when a critical value is reached. In the window of the block diagram of the LabView project, the authors created a software to command the controller and immediately execute the algorithm of the system. The connection ports are set, and the progress of the execution is displayed in the front panel window, where the measured data can be displayed, as well as the status of the devices connected to the controller. 

## 5. Results

A more accurate and professional selection of the model was carried out following thermal calculations, namely, identifying the heat loss coefficient according to Table 3, Table 4, Table 5, Table 6, Table 7 and Table 8. In this case, taking into account the values of the coefficients K2, K4–K7, the authors have adopted the average value of the loss factor 1.1.

Taking into consideration the above, the power of the air curtain will be: 126 × 25 × 1.1 = 3465 kcal/h, or 4 kW. In the case of a vestibule, the specified power can be reduced by 50–80%. Thus, at a voltage of 220 V, for a doorway 3 m wide, 3.5 m high, the suitable models are: Wing W150 with a water heating feature, or Wing C150, without water heating. On the other hand, when a 380 V power supply is available, the model Wing E150 with adjustable electric heating power is suitable. 

**Figure 7:**
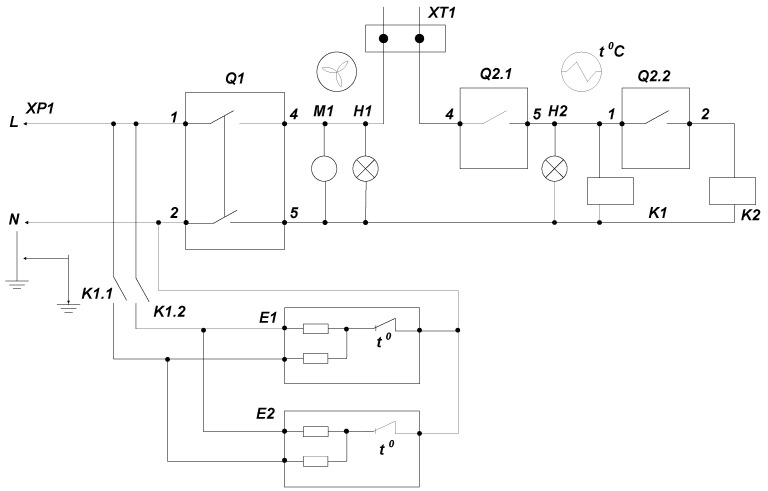
Schematic diagram of the air curtain. M1—electric motor; Q 2.1, Q 2.2—automatic switches; K1, K2—electromagnetic relays (magnetic contactors); H1, H2—signal lamps; K21, K22—electric keys; ХТ1—remote control; XP1—AC mains.

The length of the air stream, thanks to the effective fan, is up to 3.7 m for curtains with heating, and up to 4 m for models without heating. A schematic diagram of the air curtains is presented in Figure 7. In this diagram of the following models of the air curtain, AO EVR 3.0/0.3P and AO EVR 4.0/0.3P have a power of 1500 W and 2000 W, respectively. Their air consumption is 300 m^3^/h. 

**Figure 8:**
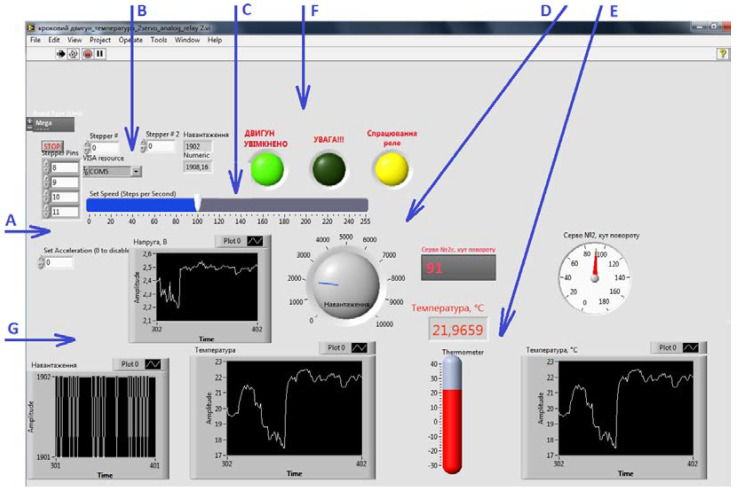
Control system interface. A—initial parameter settings; B—peripheral device connection to the COM port; C—air velocity control; D—load control; E—temperature control; F—signal controls; G—graphs of load, voltage, and temperature changes inside and outside the room.

Figure 8 shows the graphs displayed by the software, showing the dynamics of temperature changes according to the presented algorithm. The graph shows that the temperature initially rises and stabilizes at 10 °C, which corresponds to the specified permissible range of values from 8 to 12 °C. Thus, the system is operational and allows control of the indoor temperature using an air curtain. 

## 6. Discussion and Prospects

As shown by the research results, one of the most promising and up-to-date directions of energy conservation among the solutions supporting microclimate in livestock buildings is the proposed air curtain system. 

The mode of operation, based on matching the length of the air stream to the height (width) of the door is justified. With the horizontal installation of the air curtain at a height of 3.7 m, air velocity at the outlet is 9.6–11 m/s, and at the floor level, 2.3 m/s. For a guaranteed overlap, the door air curtain should be 10 cm longer than the door width, and the air curtain power should be 3465 kcal/h, or 4 kW. The use of air curtains reduces the consumption of thermal energy to maintain an optimal microclimate in livestock buildings by 10–15%. 

Please note that installing a tambour door enables a reduction of the power of the air curtain two-fold, because the heat loss through the door opening or the gate is much smaller. With the ability to regulate air curtain efficiency, it is convenient to select the model to match specific external conditions, for warmer or colder weather.

The proprietary air curtain series Wing by a renowned Polish manufacturer VTS EUROHEAT includes models from 1 to 2 m wide, with water or electric heat exchanger, as well as models without heating. All models are versatile, and can be mounted vertically or horizontally. When equipped with the original WING controller, the fan speed is controlled and the heat output is selected for models with electric heating. AC (alternating current) or DC (direct current) motors ensure low fan noise. Water-heated models with a servo-operated valve have two operating modes: with or without heating on.

Research prospects in this area include using IoT technology to monitor and control microclimate in agricultural facilities using the online-controlled air curtain. The authors have experience in implementing this technology to control other processes in agriculture, as described in [17,40]. This requires using an integrated board that allows transmitting an online signal using WiFi, Ethernet or GPRS, as well as improving the algorithm and upgrading the program in LabView. As a result, the process will be controlled not only using a desktop, but also an Android mobile device. The flexibility of the LabView software allows use of the developed installation for a broader range of tasks and various rooms with distributed parameters. Another promising factor is the use of robotic electronic devices to control the damper; examples of projects for the agricultural industry are given in [41].

An automated microclimate control system in an agricultural facility with air curtain and a SCADA system interface allow rational maintenance of a microclimate for animals and plants. The proposed solution can be used in other types of agricultural facilities. 

The presented work combines the novelty of not only technological, but also IT solutions for the use of an air curtain device, as well as software and hardware to increase energy efficiency and improve the quality of animal husbandry, and therefore the quality of animal products.

## 7. Conclusions

The presented method for effective management of the microclimate of a livestock building uses a combination of two approaches: technological and IT-based. The technological approach is the use of an air curtain, the power of which is selected according to the parameters of the room, as well as the effect of the difference between external and internal temperatures on the power of the device. On the other hand, the IT-based approach consists of using a software and hardware set based on an integrated Arduino board and a proprietary control software created using the LabView software. In contrast to the available research, the authors of this manuscript performed statistical processing of data on the calculated power of the air curtain, which depends on the difference between the internal and external temperatures. An electrical schematic diagram of the air curtain’s control was developed, as well as an experimental installation. The obtained results constitute a scientific foundation for studying the energy efficiency of agricultural enterprises. In future papers, the authors plan to formulate an energy efficiency criterion and solve the efficiency optimization problem. The fact that no IoT-based remote microclimate control has been developed is a drawback of this study. However, the authors plan to develop it in the future.

Thus, these studies confirm the initial hypothesis that the use of the described innovation increases the energy efficiency of the livestock building by at least 10%.

## Figures and Tables

**Table 1:** Structure of power consumption for milk production on farms for a headage of 200 in intensive and free-range production.

Type of Power Cost	Meaning			
	Intensive Milk Production Technologies		Free-Range Milk Production Technologies	
	Energy Expense, GJ	Share of Total Energy Expense, %	Energy Expense, GJ	Share of Total Energy Expense, %
Watering animals	72.9	1.2	72.9	1.2
Milking	268.1	4.4	608.5	9.9
Heating water	717.5	11.9	614.9	10
Primary processing of milk	259.9	4.3	259.9	4.2
Ensuring microclimate	2221.6	36.8	2129.9	34.5
Manure removal	250.5	4.2	180.9	2.9
Preparation of feed mixtures	1949.4	32.3	1998.2	32.4
Lighting	281.3	4.6	285.8	4.6
Other operations	15.9	0.3	15.9	0.3
Total	6037.1	100	6166.9	100

**Table 2:** Data on the measurements of the external and indoor temperatures of the livestock building.

External Temperature, °C	Height from the Floor, m	Indoor Temperature in the Vestibule, °C	ΔT, °C	K	N, kcal	N1, W
−20	3.9	12.9	33.5	1.1	4643.1	5.240519
−20	3	12.1	32	1.1	4435.2	5.005869
−20	1.4	10.9	31	1.1	4296.6	4.849436
−20	0.45	9.5	30	1.1	4158	4.693002
−20	−0.25	8.1	28	1.1	3880.8	4.380135
0	3.9	10.8	11	1.1	1524.6	1.720767
0	3	9.9	10	1.1	1386	1.564334
0	1.4	8.4	8.5	1.1	1178.1	1.329684
0	0.45	7.9	8	1.1	1108.8	1.251467
0	−0.25	6.9	7	1.1	970.2	1.095034
20	3.9	24.7	5	1.1	693	0.782167
20	3	22.9	3.5	1.1	485.1	0.547517
20	1.4	22.1	2.5	1.1	346.5	0.391084
20	0.45	20.9	1.5	1.1	207.9	0.23465
20	−0.25	19.9	0	1.1	0	0
33	3.9	30.1	−4	1.1	−554.4	−0.62573
33	3	29.5	−4	1.1	−554.4	−0.62573
33	1.4	28.5	−4	1.1	−554.4	−0.62573
33	0.45	27.8	−6	1.1	−831.6	−0.9386
33	−0.25	24.5	−8	1.1	−1108.8	−1.25147

**Table 3:** Values of coefficients that take into account characteristics of buildings and indoor temperature (K1 and K2).

Windows	Three-Part, Double-Glazed Window	Two-Part, Double-Glazed Window	Regular, Double Glazed Window
К1	0.85	1	1.27
Walls	Good insulation	Ц (2), heat-insulation (150 mm)	Poor insulation
К2	0.85	1	1.27
Total	6037.1	100	6166.9

**Table 4:** Values of coefficients that take into account characteristics of buildings and indoor temperature (K3).

The Window–Floor Area Ratio	10%	11–19%	20%	21–29%	30%	31–39%	40%	50%
К3	0.8	0.9	1	1.1	1.2	1.3	1.4	1.5

**Table 5:** Values of coefficients that take into account characteristics of the premises and indoor temperature (K4).

External Temperature	under −10	−10	−15	−20	−25	−30	−35
К4	0.7	0.8	0.9	1	1.1	1.2	1.3

**Table 6:** Values of coefficients that take into account characteristics of buildings and indoor temperature (K5).

Number of Walls Facing Courtyard	1	2	3	4
К5	1	1.11	1.22	1.33

**Table 7:** Values of coefficients that take into account characteristics of buildings and indoor temperature (K6).

Type of Room	Heated Roof	Warm Roof	Cold Roof
К6	0.82	0.91	1

**Table 8:** Values of coefficients that take into account characteristics of buildings and indoor temperature (K7).

Room Height	2.5 m	3 m	3.5 m	4 m	4.5 m
К7	1	1.05	1.1	1.15	1.2

## Data Availability

Not applicable.
